# Editorial: Immunology of machine perfused organs and tissues

**DOI:** 10.3389/fimmu.2022.1104268

**Published:** 2022-12-06

**Authors:** Theresa Hautz, Gerald Brandacher, Stefan Schneeberger

**Affiliations:** ^1^ organLife Laboratory, Department of Visceral, Transplant and Thoracic Surgery, Center of Operative Medicine, Medical University of Innsbruck, Innsbruck, Austria; ^2^ Vascularized Composite Allotransplantation (VCA) Research Laboratory, Department of Plastic and Reconstructive Surgery, Johns Hopkins School of Medicine, Baltimore, MD, United States

**Keywords:** organ transplantation, machine perfusion, extended criteria donor, immunology, inflammation, ischemia reperfusion injury, immunomodulation, regeneration

The clinical realization of extracorporeal, machine-based organ and tissue preservation opens a novel chapter in medicine. This technology allows for *ex-situ* organ/tissue perfusion, while still mimicking an *in-vivo* physiologic environment, in order to bridge the time between organ recovery and transplantation. It further minimizes the duration of ischemia and allows for real time assessment of organ viability, quality, and function during this state (1, 2). Most importantly, this innovative technology provides the unique possibility of organ/tissue reconditioning, immunomodulation, regeneration, and treatment outside the human body (3).

However, the impact of *ex-situ* machine perfusion on the immunological status of an organ is still largely unknown. Advances in multi-color flow cytometry, next-generation sequencing technologies, and platforms at single-cell resolution have recently opened new opportunities to better characterize intragraft cell compositions and provide novel insights into immune responses in machine perfused organs.

This Research Topic provides a collection of articles highlighting immune responses during *ex-situ* machine perfusion of organs. Original research articles include an array of studies carried out in human donor organs, as well as in pre-clinical large (porcine) and small (rat) animal models providing exciting data on the impact of machine perfusion on organ immune status and antigenicity under various conditions. In addition, this Research Topic includes relevant and interesting review articles summarizing the existing literature on immune activation during organ machine perfusion.

The concept of extracorporeal circulation has originally been established as a tool for cardiothoracic surgery, which has subsequently been extended to isolated machine perfusion of (thoracic) organs. The review article by Hatami et al. gives an overview about the activation of inflammatory and oxidative stress responses during extracorporeal live support systems (ECLP) and *ex-situ* organ perfusion (ESOP) and presents the underlying mechanisms. It further discusses possible therapeutic interventions during ECLP and ESOP to ameliorate inflammation and oxidative stress. The technology of organ machine perfusion was introduced to not only limit ischemic time of organs prior to transplantation, but also to reduce ischemia/reperfusion injury (IRI)-associated inflammation and cell death contributing to delayed graft function (DGF) or early allograft dysfunction (EAD). Panconesi et al. offer a review focusing on the impact of different machine perfusion approaches on IRI-associated responses in the liver. In this context, the authors also describe the subcellular processes and proinflammatory downstream mechanistic effects of IRI. The authors furthermore highlight protective mechanisms observed for different perfusion approaches and discuss possible treatment strategies and the delivery of specific agents to modulate posttransplant inflammation. Knijff et al. review the impact of hypothermic machine perfusion (HMP), thereby focusing on how HMP ameliorates IRI in kidney and liver transplantation. In a study of normothermic machine perfusion (NMP) including six discarded human donor livers, Lee et al. provide first insights into changes of the immune profile for up to six hours of NMP. Time-dependent, dynamic changes were observed for individual leukocyte subsets detected in liver tissue and perfusate during NMP, while cytokine levels continuously increased. These observations suggest that NMP significantly alters the immunogenicity of *ex-situ* perfused organs. Jennings et al. showed that oxygen carriers used in a rat model of normothermic *ex-situ* liver perfusion affect the phenotype of liver-resident immune cells. Interestingly, the synthetic hemoglobin-based oxygen carrier, Oxyglobin, revealed the lowest level of immune cell activation and allogenic proliferation compared to human or rat packed red blood cells (RBC), and was hence stated as the “optimal oxygen carrier” for liver NMP. The role of the hepatic immune cell repertoire in inflammatory conditions and IRI is further discussed in a review by Fodor et al. It highlights the detrimental but also regenerative potential of various immune cell subtypes, and how specific immune cell functions need to be considered when actively modifying the immune status of donor livers during machine perfusion. The mini-review by Langford et al. expands the technology of *ex-situ* organ perfusion beyond immunomodulation and suggests it as a useful tool to deliver therapeutic agents in order to rehabilitate/repair diseased and extended criteria organs of e.g. obese donors, which in the long-run could help to increase the number of transplantable organs.

Mellati et al. advocate NMP of kidneys as a suitable preservation technique and a model of reperfusion to study inflammation and immune activation when testing for novel therapies. In their study, the authors assessed the impact of alpha-1 antitrypsin (AAT) on IRI and inflammation in porcine kidneys subjected to cold static storage, +/- NMP, followed by normothermic reperfusion. Utilizing a model of NMP in porcine and human kidneys, Jager et al. investigated activation of the complement cascade. The authors observed complement activation products in the perfusate during kidney NMP, which was associated with increased levels of pro-inflammatory cytokines and a reduced creatinine clearance, and hence suggest inhibition of complement as a promising strategy to diminish kidney graft injury during NMP. Hosgood et al. analyzed the effect of free heme during *ex-situ* NMP of discarded human donor kidneys. The authors conclude that a great amount of heme was detected in the perfusate, especially when older RBCs were used. In summary, genes associated with apoptosis, inflammation and oxidative stress were upregulated during NMP, but no correlation with free heme was found.

Finally, we hope that this special Research Topic will contribute to further advance the knowledge in the field of *ex-situ* machine perfusion and its impact on graft immunogenicity. In the future, this innovative platform may provide a unique opportunity in order to create an immunologically “masked” organ/tissue, hence reducing the risk of rejection and eventually systemic immunosuppression levels. Replacement of donor cells with patient-specific, non-immunogenic cells could allow for the generation of intra organ/tissue chimerism, which might further reduce alloimmunity. All these exciting new opportunities for *ex-situ* organ manipulations may pave the way for the realization of “personalized organs” for transplant in the future.

## Author contributions

TH, GB and SS contributed to this article by providing substantial, direct and intellectual input. All authors approved the submitted version.
